# Persistierende Riechminderung nach COVID-19 – Empfehlungen der Arbeitsgemeinschaft Olfaktologie und Gustologie der Deutschen Gesellschaft für Hals-Nasen-Ohren-Heilkunde, Kopf- und Hals-Chirurgie e. V.

**DOI:** 10.1007/s00106-023-01368-w

**Published:** 2023-10-06

**Authors:** Constantin A. Hintschich, Antje Wege-Lüssen, Önder Göktas, Boris A. Stuck, Christian A. Müller, Thomas Hummel

**Affiliations:** 1 Klinik und Poliklinik für Hals-Nasen-Ohren-Heilkunde, Universitätsklinikum Regensburg, Franz-Josef-Strauß-Allee 11, 93053 Regensburg, Deutschland; 2 Hals-Nasen-Ohrenklinik, Universitätsspital Basel, Basel, Schweiz; 3 HNO Zentrum am Kudamm, Berlin, Deutschland; 4 Klinik für Hals‑, Nasen- und Ohrenheilkunde, Kopf- und Hals-Chirurgie, Universitätsklinikum Marburg, Philipps-Universität Marburg, Marburg, Deutschland; 5 Universitätsklinik für Hals‑, Nasen- und Ohrenkrankheiten, Kopf- und Halschirurgie, Medizinische Universität Wien, Wien, Österreich; 6 Interdisziplinäres Zentrum für Riechen und Schmecken, Universitätsklinikum Carl Gustav Carus, Dresden, Deutschland

**Keywords:** Riechen, Olfaktion, Hyposmie, Parosmie, COVID-19, Smell, Olfaction, Hyposmia, Parosmia, Coronavirus disease 2019

## Abstract

Der Artikel soll die existierende Literatur zu mit COVID-19 („coronavirus disease 2019“) assoziierten Riechstörungen nicht vollständig aufarbeiten, sondern die für die HNO-ärztliche Praxis relevanten Forschungserkenntnisse zusammenfassen sowie Empfehlungen zur Diagnostik und Therapie bei persistierenden Riechstörungen nach COVID-19 geben.

## Abstract (en)

This article does not intend to comprehensively review the existing literature on coronavirus disease 2019 (COVID-19)-associated smell disorders, but aims to summarize scientific evidence for otorhinolaryngological practice and provide recommendations for diagnosis and treatment of persistent smell disorders following COVID-19.

## Riechminderungen als wichtiges Symptom von COVID-19

Während der ersten beiden SARS-CoV-2-Pandemiewellen waren Riechminderung und (in vielen Fällen lediglich subjektive) Schmeckminderung unter den häufigsten Symptomen von COVID-19 („coronavirus disease 2019“) [1–3]. Hyposmien wurden in bis zu 89 % der Fälle angegeben [4], wobei in drei großen Metaanalysen durchschnittliche Prävalenzen zwischen 39 und 47 % [5–7] ermittelt wurden. Diesen drei Publikationen lagen lediglich subjektive Patientenangaben zugrunde. Jedoch korreliert die Selbsteinschätzung der Patienten nur schwach mit den Ergebnissen von psychophysischen Tests. Häufig unterschätzen oder überschätzen Patienten Veränderungen der eigenen Riechfähigkeiten [8–10]. Insgesamt ist eher von einer höheren Zahl psychophysisch bestätigter Hyposmien bei COVID-19 auszugehen [6].

Als besonders charakteristisch für Hyposmien durch eine SARS-CoV-2-Infektion, zumindest bis vor Auftreten der SARS-CoV-2-Variante Omikron, gilt das Fehlen weiterer nasaler Symptome [5]. Dadurch kann häufig eine COVID-19-assoziierte Riechminderung von Hyposmien infolge anderer viraler Infektionen der oberen Atemwege bereits anamnestisch abgegrenzt werden. Rhinoviren, Adenoviren und Influenzaviren führen häufig zu einer Schleimhautschwellung und -sekretion, die sich v. a. als Nasenatmungsbehinderung, Rhinorrhö und (konduktive) Riechminderung bemerkbar machen [5].

## Veränderung der olfaktorischen Symptome bei neueren Virusmutationen

Das ursprüngliche Wildtyp-Virus wurde von so genannten „variants of concern“ (VoC) abgelöst, die durch ihre Übertragbarkeit, ihre Virulenz und ihre Suszeptibilität einen evolutionären Vorteil haben. Den Varianten Alpha und Delta folgte die Variante Omikron mit ihren sich weiterentwickelnden Subvarianten. Nicht nur die allgemeine Morbidität und Mortalität, sondern auch viele Symptome sind bei den späteren Virusvarianten weniger schwerwiegend [1]. In Analogie dazu wurde ebenfalls eine Abnahme der Prävalenz von olfaktorischen Symptomen beobachtet [1, 11]. Eine kürzlich veröffentlichte Metaanalyse verglich die Prävalenz subjektiver Riechminderung zwischen den dominanten Virusvarianten: Im Vergleich zum SARS-CoV-2-Wildtyp lagen die Odds Ratios für die VoC Alpha, Delta und Omikron bei 0,50; 0,44 bzw. 0,18 [12]. Die Prävalenzabnahme von Riechminderungen in den neueren Virusvarianten konnte auch in verschiedenen psychophysischen Studien bestätigt werden [13–15].

## Persistenz von Riechminderungen nach COVID-19

Nach der akuten Infektion erholt sich das Riechvermögen in der Mehrzahl der Fälle innerhalb von wenigen Wochen [16], jedoch beklagen zahlreiche Patienten eine weniger rasche oder ganz ausbleibende Besserung [17]. In einer Metaanalyse wurde gezeigt, dass 5 % der Patienten, die während der Akutphase von COVID-19 von einer Hyposmie betroffen waren, noch sechs Monate später an einer subjektiven Riechminderung litten [18]. Wie bereits oben erwähnt, könnte dieser Wert noch höher sein, wenn das Riechvermögen nicht nur subjektiv eingeschätzt wird. Mittels psychophysischer Tests können gerade mit der Riechschwellenbestimmung geringe Defizite erfasst werden, die die Patienten häufig subjektiv nicht bemerken.

Die Prognose für eine Erholung der Riechfunktion ist für Frauen und für Patienten mit initial schweren Verläufen schlechter [18]. Zudem ist eine chronische Hyposmie nach COVID-19 eines der zehn häufigsten Symptome des Post-COVID-19-Syndroms [19]. So haben 3,4 % der Patienten, bei denen im zweiten Quartal 2021 ein Post-COVID-19-Syndrom diagnostiziert wurde, Störungen des Riechens und/oder Schmeckens angegeben [20]. Eine Hyposmie ist mit einer verminderten Lebensqualität assoziiert [21, 22], die sich allerdings nicht von der Lebensqualitätsverminderung unterscheidet, wie sie bei anderen postviralen Riechstörungen mit beobachtet wird [23].

## Parosmien als Zeichen einer Regeneration des Riechvermögens

In der Akutphase von COVID-19 werden Parosmien, also qualitative Riechstörungen, bei denen olfaktorische Reize verändert wahrgenommen werden, in 7 bis 27 % der Fälle beschrieben [24]. In den Folgemonaten nimmt deren Prävalenz jedoch deutlich zu [24, 25] und wird sechs Monate nach der akuten Infektion bei über 40 % der Patienten beobachtet [26, 27]. Dabei sind Frauen und junge Patienten stärker betroffen [27, 28]. Wie quantitative Dysfunktionen sind auch Parosmien mit einem negativen Einfluss auf die Lebensqualität assoziiert [27, 28].

Parosmien werden mehrheitlich als Zeichen einer Regeneration des Riechvermögens gewertet [29]. So ist eine Parosmie prognostisch günstig und mit einer ausgeprägteren Erholung der Riechfunktion assoziiert [30]. Insgesamt ist die Parosmie meist eine temporäre Erscheinung, die sich innerhalb von 6 bis 18 Monaten weitgehend bessert oder vollständig verschwindet [31, 32].

## Diagnostik

**Figure 1:**
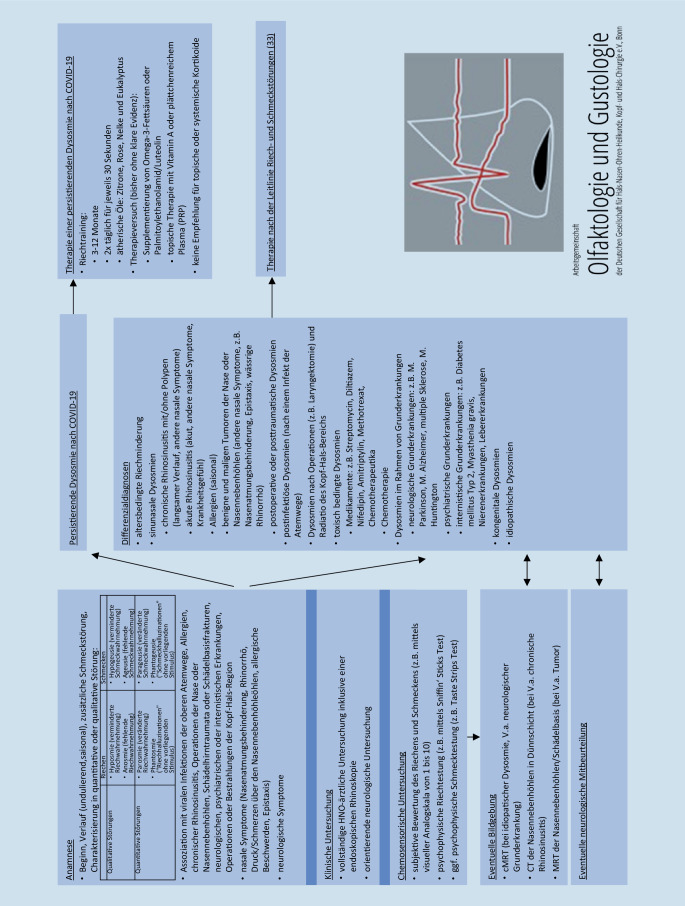

Das diagnostische Vorgehen entspricht der Diagnostik, die auch bei Riechstörungen anderer bzw. unklarer Ätiologie empfohlen wird ([33]; Abb. 1). Vor der eigentlichen HNO-ärztlichen Untersuchung sollte die sorgfältige Anamnese stehen [34, 35]. Hierbei sollte nicht nur nach olfaktorischen, sondern auch nach gustatorischen Symptomen gefragt werden. Diese werden in quantitative (Hyposmie, Anosmie bzw. Hypogeusie, Ageusie) und qualitative Störungen (Parosmie, Phantosmie bzw. Parageusie, Phantogeusie) unterteilt. Zudem sollten der zeitliche Verlauf und – abgesehen von akuten Infektionen der oberen Atemwege – mögliche andere auslösende Faktoren wie chronische Nasennebenhöhlenentzündungen, frühere Operationen, Bestrahlungen oder Traumata im Kopf- und Halsbereich sowie systemische, neurologische und psychiatrische Vorerkrankungen abgefragt werden.

Bei allen Patienten mit persistierender COVID-19-assoziierter Riechminderung sollte eine vollständige HNO-ärztliche Spiegeluntersuchung inklusive endoskopischer Rhinoskopie durchgeführt werden. So können sich Hinweise auf eine chronische Rhinosinusitis oder auf einen benignen oder malignen Tumor der Nasenhaupt- und -nebenhöhlen ergeben. Bei dem Verdacht auf eine zugrunde liegende neurologische Erkrankung sollte die neurologische Abklärung erfolgen [34]. Falls keine zugrunde liegende Ätiologie ausgemacht werden kann, sollte eine Bildgebung mittels cMRT veranlasst werden [34].

Für die eigentliche Prüfung des Riechvermögens gibt es eine Vielzahl etablierter Methoden: Die einfachste Abschätzung kann durch den Patienten selbst erfolgen. Hierbei wird nach einer subjektiven Selbsteinschätzung des Riechvermögens gefragt, wobei sich die Angabe auf einer visuellen Analogskala von 1 bis 10 bewährt hat.

Präziser im Vergleich zu Selbsteinschätzungen sind psychophysische Riechtests [36]. Hier bietet sich der so genannte Sniffin’ Sticks Test an, bei dem Düfte mittels wiederverwendbarer Filzstifte dargeboten werden [37]. Es werden verschiedene Varianten unterschieden: Bei dem so genannten SDI-Test werden neben der Geruchsidentifikation (I) auch die Riechschwelle (S) und die Geruchsdiskrimination (D) getestet. Daneben gibt es auch weniger aufwendige Screeningtests, die i. d. R. nur aus einem Identifikationstest bestehen und bei denen die Testung nur wenige Minuten in Anspruch nimmt.

Ein besonderer Vorteil der psychophysischen Testung mittels Sniffin’ Test ist die Validierung für eine wiederholte Testung [38]. Somit lässt sich die olfaktorische Funktion im Langzeitverlauf besonders gut quantifizieren, was gerade bei persistierenden bzw. sich nur langsam bessernden Hyposmien nach einer SARS-CoV-2-Infektion bedeutsam ist.

## Therapie von Riechstörungen nach COVID-19

Bisher sind nur wenige Therapiemöglichkeiten bei persistierender Hyposmie nach COVID-19 verfügbar (Abb. 1). Eine Reihe von Studien konnten zeigen, dass ein Riechtraining zu einer signifikanten Besserung des subjektiven und psychophysisch erfassbaren Riechvermögens führt [39–41]. Für das Riechtraining sollte die Patientin/der Patient zweimal täglich für etwa 30 s an jeweils vier verschiedenen Duftquellen riechen. Als „Trainingsdüfte“ haben sich Zitrone, Rose, Nelke und Eukalyptus, z. B. in Form von Duftölen, bewährt [42]. Insgesamt sollte das Training über einen Zeitraum von 3–12 Monaten durchgeführt werden, wobei nach 3–4 Monaten die Duftqualitäten geändert werden sollten.

Zudem gibt es interessante Therapieansätze mit einer Supplementierung von Omega-3-Fettsäuren [43] oder Palmitoylethanolamid/Luteolin [44] und der topischen Applikation von plättchenreichem Plasma (PRP) [45, 46] bzw. Vitamin A in die Riechrinne [47]. Hierbei müssen weitere Studien die bisherigen positiven Effekte jedoch überprüfen. Die Behandlung mit intranasalen Kortikosteroiden scheint bei postviralen Riechstörungen keine positive Wirkung auf die Verbesserung der Riechfunktion zu haben [48–50]. Auch für systemische Kortikosteroide sind die Ergebnisse bisher inkongruent [51, 52], sodass aufgrund der möglichen Nebenwirkungen deren Anwendung nicht generell empfohlen wird [53].

## Fazit für die Praxis

- Verglichen mit dem ursprünglichen Wildtyp-Virus sind später aufgetretene Varianten von SARS-CoV‑2 signifikant seltener mit einer Riechminderung assoziiert.
- Die Riechfunktion erholt sich in den meisten Fällen innerhalb von Wochen, jedoch berichten ungefähr 5 % der ursprünglich betroffenen Patienten noch sechs Monate nach der akuten Infektion von einer Symptompersistenz.
- Die Untersuchung sollte neben einer ausführlichen Anamnese eine vollständige HNO-ärztliche Untersuchung einschließlich endoskopischer Rhinoskopie und eine psychophysische Testung der Riechfunktion umfassen.
- Bisher konnte lediglich für das konsequente und langfristige Riechtraining eine Verbesserung der Riechfunktion überzeugend nachgewiesen werden. Eine Therapie mit topischen oder systemischen Kortikosteroiden wird bei persistierenden Riechminderungen nach COVID-19 nicht empfohlen.
